# Emergency online teaching during COVID-19 pandemic versus conventional offline methods in morphology experiment: An observational comparative study

**DOI:** 10.1097/MD.0000000000043649

**Published:** 2025-08-01

**Authors:** Yanyan Qiu, Jiaying Fan, Qing Xia, Sunhong Chen

**Affiliations:** a Center for Experimental Medical Science Education, Shanghai Jiao Tong University School of Medicine, Shanghai, China.

**Keywords:** COVID-19, evaluation, experimental course, Morphology, Online teaching

## Abstract

The Coronavirus Disease 2019 (COVID-19) pandemic prompted global universities to adopt online teaching, yet limited research exists on its efficacy in morphology experiments. This study evaluates online versus offline teaching outcomes in histology and pathology courses during 2020 to 2023. We implemented hybrid (asynchronous/synchronous) online morphology experiments and compared results with historical offline face-to-face teaching. Outcomes were assessed through lab reports, mid-term/final exams, and a validated satisfaction survey (5-point Likert scale). Among 855 participants (446 online, 409 offline), 55.83% preferred offline teaching for enhanced interaction (*P* < .01), though online learners reported comparable satisfaction (4.25 ± 0.64 vs 4.38 ± 0.84, *P* = .157). Online groups scored lower in lab reports (19.37 ± 1.50 vs 19.63 ± 1.39, *P* < .001) and mid-terms (25.83 ± 3.41 vs 27.54 ± 3.07, *P* < .001), but achieved parity in finals (39.43 ± 8.63 vs 40.57 ± 7.51, *P* = .112). All of these results differed in different grades, genders or specialties. The online teaching mode of morphology experiments produces comparable teaching effects, especially during the COVID-19 epidemic. It also provides additional experience, new perspectives, and new ideas for offline teaching. It indicated combining online flexibility with in-person engagement could enhance future teaching outcomes.

## 1. Introduction

In December 2019, the Coronavirus Disease 2019 (COVID-19) pandemic started, and spread all around the world rapidly. The pandemic had multiple influences on our life including higher education. Many colleges and universities had to conduct online teaching to maintain the continuity of education in these 3 years.^[1,2]^ This massive unplanned transition from face-to-face teaching to an exclusively online teaching presented challenges for universities, especially for medical schools where teaching was conducted through large amounts of practical activities and laboratory exercises.^[3]^

Online teaching includes synchronous and asynchronous mode. Synchronous teaching requires “live” broadcasting, such as teleconferencing and videoconferencing, and asynchronous teaching allows delayed broadcasting, such as e-mail, recorded videos and discussion forums, etc.^[4]^ Each form has its advantages and disadvantages. Live broadcasting can provide more efficient and real-time interactions and personal engagement. Meanwhile, it requires a lot of equipment and networks for educators and students, especially for the students in remote areas. On the contrary, asynchronous teaching can meet the needs of more students, and it can be more flexible than synchronous teaching. Students can study the course at anytime, anywhere, and also can study it over and over again.^[5]^

Medical morphological experiments, especially the experiments of histology and pathology are basic experimental courses for medical students and they are indispensable parts of medical education. In our center, medical morphology is an independent experimental course integrated from the experiments of histology and pathology. This course is based on body morphological changes, by observing normal and abnormal gross specimens combined with histological changes comparing normal and abnormal states. It aims to strengthen the early clinical practice and the capacity of diagnosis for students. Different from other experimental courses, medical morphology is based on observing rather than operating. Observing a large number of specimens and slides is the core of this course. Therefore, online teaching of medical morphology needs integral virtual resources, such as digital slides and specimen library, which are open access to students.

In the past decade, a whole pathology gross specimen imaging library and a virtual microscopy-based platform had been introduced. Students could study on their iPads (large screens) and mobile devices (small screens) anywhere online independently. Both specimen library and virtual slides platform do not require a dedicated browsing software, and can be easily viewed through webpage access. Within the specimen library and virtual slides platform, all the students could browse the same gross specimen or virtual slide at the same time. Students could also use the “drag,” “rotate,” and “zoom in or out” options to view relevant slides and specimen.^[6,7]^ Even more, they can mark the key cells, structures or lesions on the gross specimen or virtual slides.

The online teaching system had greatly developed and improved during the epidemic of COVID-19. Many researches on online teaching were conducted and constructive suggestions were provided.^[8]^ However, researches on histology or pathology online teaching are limited,^[9]^ and very few studies evaluate the effectiveness of online teaching of morphological experiments. In our study, we integrated our multiple digital library, virtual microscopy platform, and Chaoxing app to meet the requirements of online-teaching and carried out medical morphology online course to meet the needs of educators and students. The purpose of this study was to evaluate the effects of online mode of medical morphological experiment. We hypothesized that online teaching would yield comparable academic performance and student satisfaction compared to offline teaching in morphology experiments.

## 2. Materials and methods

### 2.1. Research participants

The subjects participated in this study were undergraduate students in their second year or higher, including specialties in clinical medicine, 8-year clinical medicine, pediatrics and stomatology. Most of these students had learned part of the course offline, so they had mastered some of the basics. We compared students from the same medical program and academic year (offline group, n = 409 vs online group, n = 446). To minimize selection bias, cohorts were matched by academic year, specialty, curriculum structure and prior foundational courses.

### 2.2. Course design and delivery

**Figure 1.**
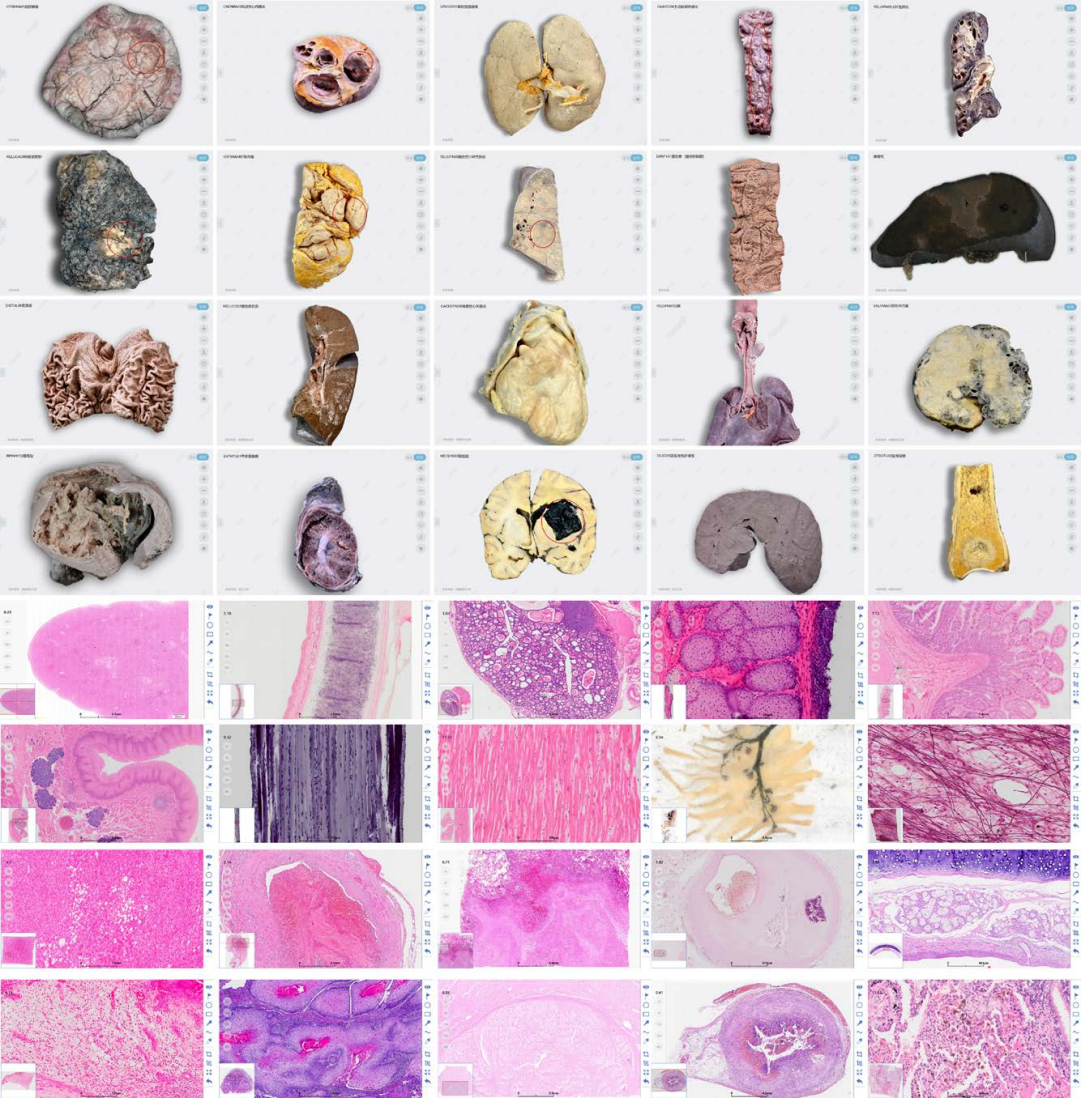
Examples of specimen library and digital slides.

The online course was designed to be consistent with the traditional offline face-to-face teaching mode. Online teaching was delivered both asynchronously and synchronously. In asynchronous teaching, high-quality teaching videos were recorded by senior and experienced teachers to explain key points and were uploaded on Chaoxing platform open to the students. In synchronous teaching, teachers used Tencent Conference app live streaming to introduce main topics, describe characteristics and answer questions. Then, students conduct the practical or microscopic part using our digital slides and specimen library (Fig. 1). They should complete assignments by drawing or taking screenshots with key structural markings and feature descriptions as what they would do in offline face-to-face teaching. In class, students could contact with teachers by WeChat, Chaoxing, or Tencent Conference app.

### 2.3. Students’ perception and data collection

The participants’ perception was obtained by a self-designed short satisfaction questionnaire via Sojump on line. The questionnaire contained a total of 15 self-assessment questions. Data were collected on grade, gender, specialty, teaching mode, preferences for online versus offline teaching and the reasons, 2 questions about teachers assessment (a 5-point Likert scale, Cronbach’s α = 0.903), 2 questions about interaction assessment (a 5-point Likert scale, Cronbach’s α = 0.729), 2 questions about learning experience evaluation (a 5-point Likert scale, Cronbach’s α = 0.911), 3 questions about homework and tests evaluation (a 5-point Likert scale, Cronbach’s α = 0.703). This study was approved by Shanghai Jiaotong University School of Medicine. Informed consent was obtained from all participants. All survey responses and academic scores were anonymized at the point of collection. Data were stored on password-protected institutional servers with access restricted to the research team. Participants were informed of their right to withdraw data within 30 days of submission.

### 2.4. Teaching evaluation and assessment

During the delivering of medical morphological experimental online course, students were required to complete 2 tests the same as offline face-to-face teaching in the middle (30 points) and at the end (50 points) of the term respectively, and submit lab reports (20 points) of every module as the same as the students who studied the course offline in their second year. The total score summarized from these 3 parts of each student could be obtained. All of the tasks and exams were conducted online. These assignments and exams allowed us to evaluate the effect of the study of medical morphological experiment in the whole process and in multiple dimensions both online and offline.

### 2.5. Statistical analysis

For continuous data such as test scores and assessment scores are presented as mean ±  standard deviation. Student *t* test, nonparametric tests or 1-way ANOVA (Analysis of Variance) were performed to compare the means between different groups using SPSS19.0 (Statistical Package for the Social Sciences, New York) software package. When the 1-way ANOVA revealed significant differences, post hoc analysis with multiple-comparison procedures using the Bonfferoni method were performed. For categorical data such as grade, gender and specialty, the percentages were used, and Chi-square test was performed. *P* < .05 was considered statistically significant.

## 3. Results

### 3.1. General information of the online teaching

**Figure 2.**
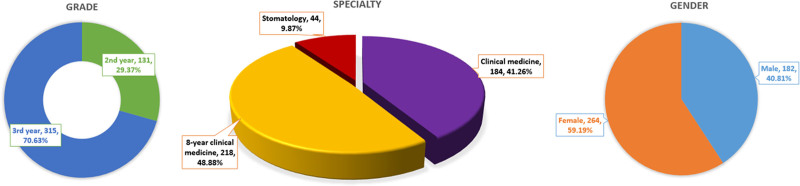
The general information of the participants in this study.

To better understand students’ preferences and perception of the online morphological experiments, we distributed a self-designed questionnaire to the participants. A total of 446 students finished the questionnaire, and all of the collected questionnaires were valid (100%). Among them, there were 315 third year students and 131 second year students. As to the specialty, they were from clinical medicine (41.25%), 8-year clinical medicine (48.88%) and stomatology (9.87%). And there were 182 males (40.81%) and 264 females (59.19%). The general data were shown in Figure 2.

### 3.2. Survey on student satisfaction of online morphological experiments

**Figure 3.**
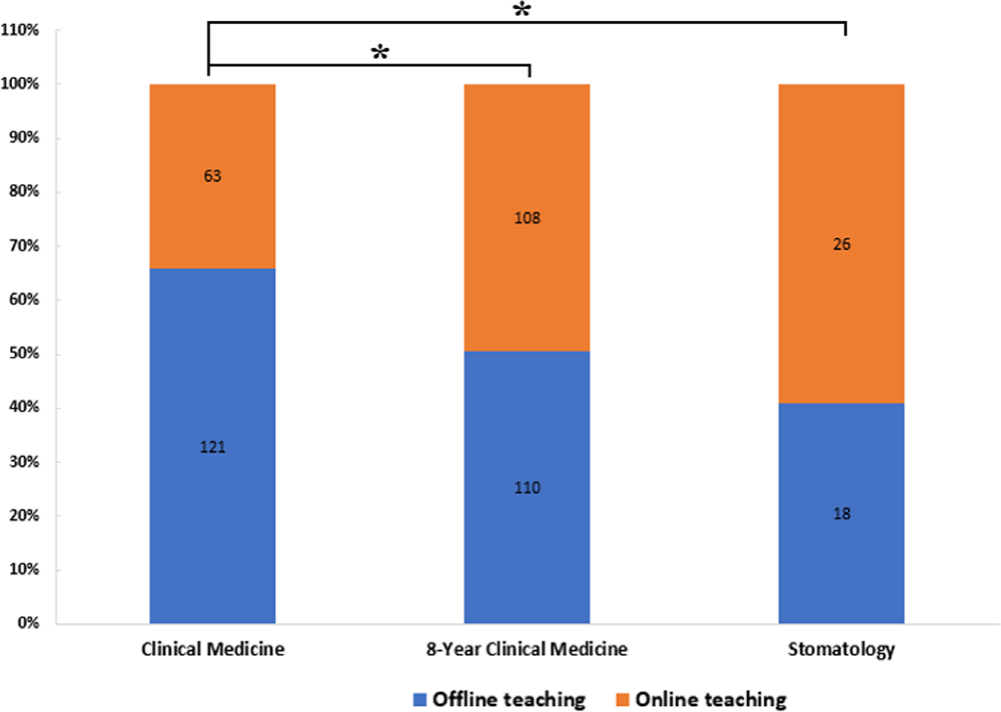
Comparison of student preferences for online vs offline teaching modes across majors. * indicates significant differences (*P* < .05, chi-square test). Error bars represent 95% confidence intervals.

Incomplete surveys (online: n = 7; offline: n = 5) and missing scores (0.6% of data) were excluded. Sensitivity analyses using median imputation showed no outcome changes. The evaluation questionnaire showed that students preferred traditional offline face-to-face teaching generally (55.83% offline vs 44.17% online), because slides and specimens in morphology laboratory were more diverse, and students could interact with teachers and classmates more efficiently. However, the preferences were influenced by multiple factors, such as grade, gender and specialty. As the results showed, the second-year students, female students and students majoring in 8-year clinical medicine and stomatology preferred online teaching, these differences were statistically significant (Table 1 and Fig. 3). The reasons might be that, students had the freedom to schedule learning time, could find teachers online at anytime, and the recorded lessons were available for them to listen to repeatedly, making it easier to understand.

**Table 1:** Students’ preference to online teaching or offline teaching, chi-square test.

Information	Preferences		Total	*χ* ^2^	*P*	φ/Cramer’s *V*	95% CIs
	Online teaching	Offline teaching					
Grade							
2nd yr	71 (54.20)	60 (45.80)	131	7.564	.006**	0.130	[0.038, 0.222]
3rd yr	126 (40.00)	189 (60.00)	315				
Gender							
Male	70 (38.46)	112 (61.54)	182	4.064	.044*	0.095	[0.003, 0.187]
Female	127 (48.11)	137 (51.89)	264				
Specialty							
Clinical medicine	63 (34.24)	121 (65.76)	184	13.881	.001**	0.177	[0.087, 0.267]
8-yr clinical medicine	108 (49.54)	110 (50.46)	218				
Stomatology	26 (59.09)	18 (40.91)	44				
Total	197 (44.17)	249 (55.83)	446				

CIs = confidence intervals.

* *P* < .05.

** *P* < .01.

We also found that 86.77% of the students were adapted to online teaching, and their adaptation to online teaching was positively correlated with whether they had participated in online learning (*r* = 0.147, *P* < .01), and with network support in the process of class (*r* = 0.17, *P* < .001). No correlation was observed between students’ adaptation to online teaching and their familiarity with morphology online resources (*r* = 0.09, *P* = .127).

Nearly 97% of the students indicated that the network access was sufficient to support online learning, they could complete and submit the lab reports on time. More than half of the students considered that the learning time, learning interest and initiative spent on online learning were similar to those spent on offline learning, but the communication and discussion with teachers or classmates were significantly reduced, which was considered as disadvantages of autonomous online learning. Meanwhile, the questionnaire revealed that more than 80% of students were satisfied with our digital virtual slides and specimen library, which they believed were abundant, convenient to use, clearly labeled, and easy to understand.

In addition, we also obtained 409 assessment scales of the course before the outbreak of COVID-19. These 409 students all received offline face-to-face teaching. We compared the assessment data between the 2 teams. Results indicated that the students from online learning team were generally satisfied with the morphology experimental lessons according to the 5-point Likert scale (4.25 ± 0.64 online vs 4.38 ± 0.84 offline, *P* = .157, Cohen’s *d* = −0.17, 95% CI [−0.35, 0.01]). However, the scores of some assessment aspects were significantly different. For example, students reported significantly higher engagement during offline face-to-face learning, and they were able to receive more attention from teachers, to interact more promptly, and to learn more efficiently. (The results were shown in Table 2).

**Table 2:** Students’ assessment to online teaching or offline teaching, 2-tailed independent *t* test.

Assessment aspects	Score (mean ± SD)		*t*	*P*	Cohen’s *d*	95% CIs
	Online teaching (n = 446)	Offline teaching (n = 409)				
Teacher’s presentation of knowledge points	4.23 ± 0.68	4.28 ± 0.59	−4.686	.214	−0.08	[−0.16, 0.01]
Teacher’s attention to students	3.93 ± 0.80	4.30 ± 0.63	−13.536	.000**	−0.51	[−0.61, −0.42]
Teacher’s tutoring and Q&A	3.98 ± 0.82	4.36 ± 0.81	−6.741	.000**	−0.47	[−0.58, −0.37]
Contact and interaction with teachers	3.79 ± 0.88	4.43 ± 0.72	−11.559	.000**	−0.83	[−0.93, −0.72]
The quantity and difficulty of homework	4.00 ± 0.76	4.14 ± 0.85	−4.451	.175	−0.17	[−0.27, −0.07]
Teacher’s feedback on homework	4.11 ± 0.74	4.03 ± 0.94	3.217	.126	0.10	[0.00, 0.20]
Learning efficiency	3.78 ± 0.82	4.30 ± 0.88	−8.775	.000**	−0.61	[−0.72, −0.51]
Learning experience and harvest	3.82 ± 0.76	4.54 ± 0.63	−15.047	.000**	−1.06	[−1.17, −0.95]
The methods and difficulty of tests	3.63 ± 0.95	4.07 ± 0.78	−7.522	.000**	−0.52	[−0.63, −0.41]

CIs = confidence intervals, SD = standard deviation.

** *P* < .01.

### 3.3. Evaluation of the teaching effect of online morphological experiments

**Figure 4.**
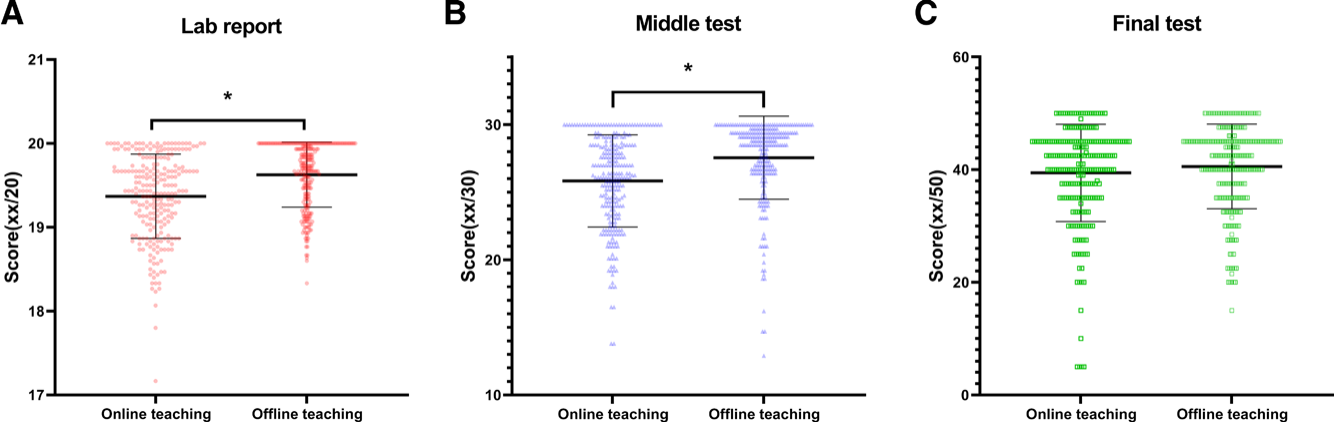
Academic performance of second-graders comparison between online (n = 243) and offline (n = 259) groups. (A) Scores of lab report; (B) scores of middle test; (C) scores of final test. * *P* < .05 (2-tailed independent *t* test).

To evaluate the teaching effects, we compared the scores of second-grade students in online teaching (n = 243) with those in offline teaching (n = 259). For the lab reports, the average score of the online teaching group was slightly lower than that of the offline teaching group (19.37 ± 1.50 vs 19.63 ± 1.39, *P* < .001, Cohen’s *d* = −0.18, 95% CI [−0.35, −0.01]). The middle test score of the online teaching group was significantly lower than that of offline teaching group (25.83 ± 3.41 vs 27.54 ± 3.07, *P* < .001, Cohen’s *d* = −0.53, 95% CI [−0.71, −0.35]). And for the final test, the average score of the online teaching group had no significant difference as compared to that of the offline teaching group (39.43 ± 8.63 vs 40.57 ± 7.51, *P* = .112, Cohen’s *d* = −0.14, 95% CI [−0.31, 0.03]). The data were shown in Figure 4.

**Figure 5.**
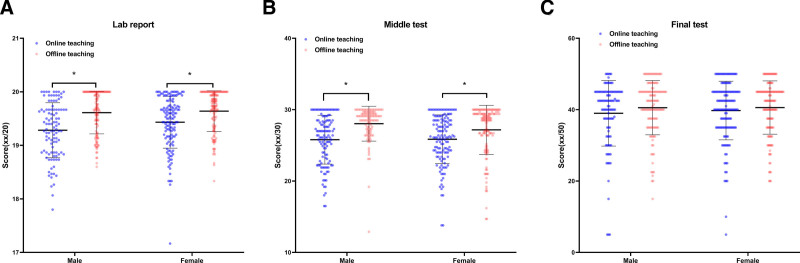
Academic performance of second-graders comparison between online and offline groups stratified by gender (Male: n = 226, online 104, offline 122; Female: n = 276, online 139, offline 137). (A) Scores of lab report; (B) scores of middle test; (C) scores of final test. * *P* < .05 (2-tailed independent *t* test).

**Figure 6.**
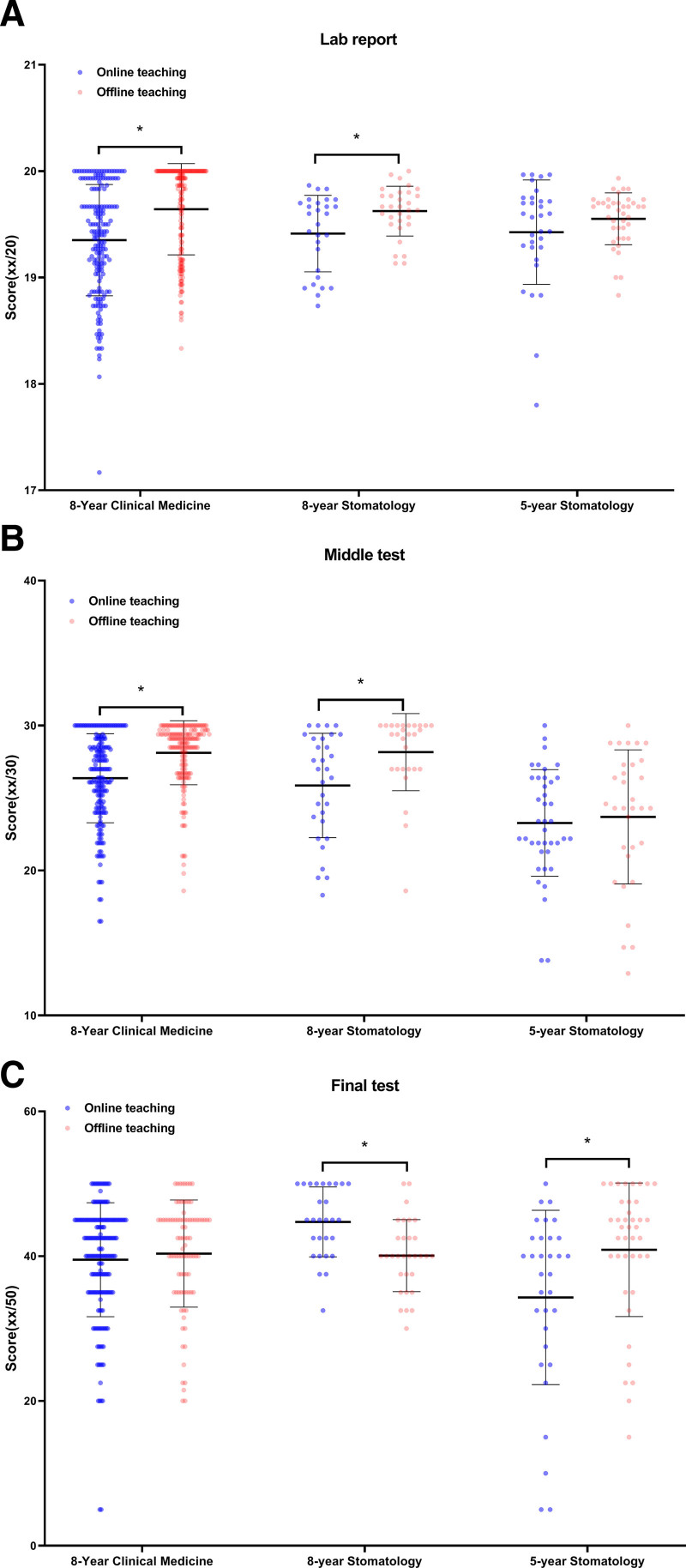
Academic performance of second-graders comparison between online and offline groups stratified by specialty (8-yr clinical medicine: n = 371, online 183, offline 188; 8-yr stomatology: n = 59, online 28, offline 31; 5-yr stomatology: n = 72, online 32, offline 40). (A) Scores of lab report; (B) scores of middle test; (C) scores of final test. * *P* < .05 (2-tailed independent *t* test).

Subsequently we stratified and analyzed the data by gender. We found the results showed a consistent trend with the overall results (Fig. 5). In addition, we stratified and analyzed the data by specialty. As the results shown, online and offline teaching groups demonstrated significant differences in the average scores of the lab report and 2 tests across the different specialties (Fig. 6).

## 4. Discussion

Since the outbreak of COVID-19 in 2020, colleges and universities all around the world have made full use of online teaching resources to actively explore and practice online teaching for more than 3 years.^[10,11]^ Scholars conducted a survey on the initial perspectives of online teaching (including theory and experiments) from 1,18,030 medical students in 90 medical colleges in China due to the impact of the COVID-19 epidemic.^[12]^ The results showed that 62.1% of students were relatively satisfied with online teaching, while 8.7% of students expressed dissatisfaction. The main problems of online learning were network congestion and poor interaction, which were more evident in experimental operational courses. Although students had given full recognition to the effectiveness of online teaching carried out during the special period of the pandemic, more students believed that offline face-to-face teaching is more effective. One research on histology online teaching showed that students’ satisfaction with theoretical courses was higher than that of virtual slides based experimental teaching. Students believed that the virtual slides platform used in online experimental teaching had some problems such as network congestion, and poor interaction with classmates and teachers.^[13]^

According to this study, students tended to prefer traditional offline face-to-face experimental teaching of microscopes over virtual slides based online experimental teaching generally. This was because they believed that offline teaching offered better real-time interaction between teachers and students and a more intuitive observation of slides. However, the trend of preference to certain subjects was influenced by gender and specialty, as seen among students of different genders and majors whose preferences varied. The analysis of students’ homework and test scores revealed that the overall teaching effect of virtual slides based online teaching is comparable to that of traditional offline teaching. However, mid-term test scores of online teaching were slightly lower than those of offline teaching, possibly due to students’ initial lack of adaptability to online teaching of experimental courses. Nevertheless, after fully adapting in the later stage, final test scores could be equal to those of offline teaching. Meanwhile, online teaching resources such as teachers’ presentation, specimen library and digital slides were much more convenient for students to review repetitively. These scores were influenced by gender and specialty. Nevertheless, the non-randomized comparison may introduce residual confounding, despite our efforts to match groups. And unmeasured confounders, such as household income or prior digital literacy, may influence outcomes. However, mandatory institution-wide online transition during COVID-19 reduced self-selection bias. Future studies should prioritize randomized controlled trials when feasible.

The effectiveness of online teaching was largely dependent on the stability of the network and equipment performance. These factors had played a crucial role in determining students’ learning experience, enthusiasm, and overall teaching effectiveness.^[14]^ Cloud platforms such as Chaoxing enabled teacher–student interaction, however, the effectiveness of this interaction was limited to some extent by the network and equipment performance. Furthermore, students’ behavior when observing slides online was difficult to monitor, as it was dependent on their self-awareness.^[15]^ In contrast, offline teaching allowed face-to-face interaction between students and teachers, enabled real-time feedback and adjustments, which was a key advantage of offline courses that online teaching cannot surpass. Additionally, the use of virtual slides, while providing fast and diverse learning opportunities, weakened students’ ability to operate microscopes and may reduce their interest in hands-on learning. This would negatively impact learning efficiency and scientific research training. In this study, virtual slides were used as representatives of typical structures with complete shapes. However, since no more than 3 slides were selected by experts, students were not exposed to the diversification of slides from different people in different shapes or sizes. This may hinder their innovative thinking. Further, virtual slides came with prestored key structure marks which facilitated independent learning but may discourage active thinking and seeking, ultimately not promoting active learning of students. On the other hand, we believed that online teaching offered several advantages, including flexibility, resources sharing, interactivity, and visualization. Students had flexibility of choosing to study at their own pace and from any location. The use of the Internet allowed resources sharing and reduced the waste of laboratory resources.^[16]^

Our study was conducted based on our online teaching experience in recent years and feedback from students. The study outcomes suggested we should take several measures for the future morphological experimental teaching. Promoting digital resources as effective supplemental materials to experimental teaching. Promote the digitization of self-owned high-quality teaching slides and specimen, making it an abundant morphological digital resource library. Using digital library as an extracurricular supplementary content for offline teaching can facilitate students who have enough energy to expand their knowledge and effectively improve their learning efficiency. At the same time, excellent slides and specimen are stored and accumulated for a long time, and the morphological digital library is gradually enriched and continuously improved. Integrating multiple platforms to optimize the utilization of morphological digital resources. This can be achieved by integrating digital resources and lesson recording videos into a single platform, which will facilitate real-time communication among teachers and students. Additionally, opening an experimental report upload and feedback channel. These measures will enhance students’ sense of learning achievement and happiness, ultimately improve their self-learning ability. To support these initiatives, it is important to enhance the performance of network servers. A high-speed network is a prerequisite for the popularization of digital resources. Providing comprehensive teacher training to ensure the quality of teaching. It is essential that teachers have professional knowledge, are proficient in using digital resources, and can effectively integrate online libraries with offline teaching. We believe these measures can help achieve better results in teaching activities and enhance students learning experience.

## 5. Conclusion

The online morphology experimental curriculum demonstrated comparable outcomes to traditional offline methods, nonetheless, the observed challenges such as reduced student satisfaction and lower mid-term scores, highlight its limitations, which may constrained its advancement. These mixed results suggest that online learning can serve as a viable alternative during emergencies like the COVID-19 pandemic. Future efforts should prioritize hybrid models that combine online flexibility with offline interaction strengths to optimize educational outcomes.

## Author contributions

**Data curation:** Yanyan Qiu, Qing Xia.

**Funding acquisition:** Yanyan Qiu.

**Project administration:** Yanyan Qiu.

**Resources:** Jiaying Fan.

**Software:** Jiaying Fan.

**Supervision:** Sunhong Chen.

**Writing – original draft:** Yanyan Qiu.

**Writing – review & editing:** Sunhong Chen.
